# Optimizing Cardiovascular Outcomes in Type 2 Diabetes: Early Initiation of Dapagliflozin and Sitagliptin From a Cardiologist's Perspective

**DOI:** 10.7759/cureus.81858

**Published:** 2025-04-07

**Authors:** K. Jaishankar, Rajeev Garg, Abhijit Kulkarni, Johann Christopher, Ravindran R, Peeyush Jain, Pankaj Sarkar, Vivek Mahajan, Sunil Sathe, Lachikarathman D, Abhijit Pednekar, Ashish Prasad, Rohan Kesarkar

**Affiliations:** 1 Cardiology, Medway Heart Institute, Chennai, IND; 2 Cardiology, Gleneagles Aware Hospital, Hyderabad, IND; 3 Cardiology, Apollo Hospitals, Bangalore, IND; 4 Cardiology, Dr. Malathi Manipal Hospital, Bangalore, IND; 5 Cardiology, CARE Hospitals, Hyderabad, IND; 6 Cardiology, Rays Clinic Cardiac and Cosmetic Centre, Chennai, IND; 7 Cardiology, Fortis Escorts Heart Institute, New Delhi, IND; 8 Cardiology, North City Hospital, Kolkata, IND; 9 Cardiology, Fortis Hospital, Kalyan, IND; 10 Cardiology, Dr. Sunil Sathe (Cardiac Care &amp; Counselling Centre) Clinic, Pune, IND; 11 Cardiology, Sri Jayadeva Institute of Cardiovascular Sciences &amp; Research, Bangalore, IND; 12 Scientific Services, USV Pvt Ltd., Mumbai, IND; 13 Diabetes and Endocrinology, Scientific Services, USV Pvt Ltd., Mumbai, IND

**Keywords:** cardiology, dapagliflozin, diabetes, dipeptidyl peptidase-4 inhibitors, fixed-dose combination, sitagliptin, sodium-glucose co-transporter 2 inhibitors

## Abstract

Introduction: Cardiovascular (CV) disease (CVD) risk is greater in patients with diabetes mellitus and is the major contributor to disability and premature mortality compared to those who do not have diabetes. The clinical implications of CVD in people with type 2 diabetes mellitus (T2DM) have increased the emphasis on concurrent treatment to prevent the onset of CVD through personalized management for glycemic control and CVD risk management.

Methods: Key opinion leaders, comprising 98 cardiologists from across India, participated in seven advisory board meetings held in various cities to explore the challenges and strategies for the early initiation of fixed-dose combinations (FDCs) of sodium-glucose co-transporter-2 inhibitors (SGLT2i) and dipeptidyl peptidase-4 inhibitors (DPP4i) with a focus on the combination of dapagliflozin and sitagliptin in addressing the CVD risks in patients with T2DM and high risk for CV complications. The expert group discussed the available literature evidence from the clinical trials, systematic reviews, and real-world studies on the benefits of FDC of SGLT2i and DPP4i and FDC of dapagliflozin and sitagliptin to provide rational and practical guidance for its optimal use in addressing the CVD risks in patients with T2DM.

Results: The expert group emphasized the importance of timely glycemic control and early initiation of combination therapy of FDC of SGLT2i + DPP4i in T2DM with CVD risks. Addressing multiple pathophysiological aspects of T2DM is crucial, and considering combination therapy with SGLT2i and DPP4i may be pertinent in this context. Combining dapagliflozin and sitagliptin in FDC to target multiple pathophysiological pathways for T2DM appears to have several glycemic and extra-glycemic benefits.

Conclusion: This practical guidance document provides valuable insights from leading cardiologists that would support clinicians in selecting the synergistic combination SGLT2i + DPP4i (dapagliflozin + sitagliptin) FDC as an appropriate treatment choice in early intensive therapy in managing people with T2DM and CVD risk for better patient outcomes. The expert opinion in this guidance builds on the established guideline recommendations on FDC of SGLT2i and DPP4i.

## Introduction

Cardiovascular (CV) disease (CVD) is the primary cause of disability and mortality in people with diabetes [1]. The risk of CVD is 1.5-2 times greater in people with type 2 diabetes mellitus (T2DM) compared to those who do not have T2DM. This is of great concern given the widespread prevalence of diabetes and the increasing number of elderly individuals globally [2]. A 1% increase in glycated hemoglobin (HbA1c) levels is linked to an 18% rise in the risk of macrovascular disease, whereas keeping HbA1c levels below 7% is associated with a 37% reduction in CVD risk over an 11-year period. Moreover, for every 1% increase in HbA1c levels above the range of 6%-6.9%, there is a corresponding rise in the mortality rate [1].

Achieving and sustaining glycemic targets is important to reduce the risk of complications in people with T2DM. Despite numerous treatment options available, a significant number of people with T2DM continue to have inadequate glycemic control [3]. Because of the progressive decline in β-cell function, most people with T2DM in the due course of time will need combination therapy with two or more antihyperglycemic drugs with a complementary mechanism of action [4].

The clinical burden of the consequences of CVD in people with T2DM has led to an increasing emphasis on concurrent treatment for both conditions. Significant efforts have been made to prevent the onset of CVD in people with T2DM, focusing on personalized glycemic control and the management of CV risk, with recently introduced lipid-lowering and glucose-lowering medications [5].

Sodium-glucose co-transporter-2 inhibitors (SGLT2i) have demonstrated improved CV outcomes independent of glycemic control, making them a key component in managing cardiometabolic risk in people with T2DM [6]. The SGLT2i with proven CV benefits are recommended by guidelines for people with T2DM and established atherosclerotic CVD (ASCVD); those with multiple ASCVD risk factors, diabetic kidney disease, or heart failure (HF); or those in need of weight reduction [7-9].

The use of SGLT2i in combination with dipeptidyl peptidase-4 inhibitors (DPP4i) with glycemic and extra-glycemic benefits helps to target various pathophysiological pathways in T2DM and provides a distinct advantage, supporting the early implementation of this combination in the management of T2DM [6]. The combination of SGLT2i and DPP4i results in a greater reduction in mean HbA1c (-0.62%) when compared to DPP4i used alone (-0.35%) with significant weight loss seen in the combination vs. DPP4i used alone, but not vs. SGLT2i. The risk of hypoglycemic events was low and consistent across the treatment group. The HbA1c reduction with SGLT2i/DPP4i was proportional to baseline HbA1c but was modest when compared to SGLT2i alone, regardless of baseline levels [10].

The SGLT2i/DPP4i combination therapy offers improved glycemic control and greater weight loss without increasing the risk of hypoglycemia or urinary tract infections (UTIs), compared to placebo/DPP4i in patients with inadequately controlled T2DM. This combination results in a significant reduction in HbA1c (-0.6%, 95% CI: -0.7 to -0.5%) and a decrease in fasting plasma glucose (FPG), postprandial blood glucose (PPBG), and body weight. The risk of hypoglycemia was higher with SGLT2i/DPP4i when insulin or sulfonylureas were used as background therapy. While the risk of UTIs did not increase, the addition of SGLT2i was associated with a higher incidence of genital infections [11].

The SGLT2i helps reduce hyperglycemia by increasing the urinary glucose excretion independent of insulin secretion or action, and the DPP4i inhibits the breakdown of active incretin hormones, improving glucose homeostasis by increasing insulin secretion and decreasing glucagon secretion in a glucose-dependent manner [11]. The fixed-dose combination (FDC) of SGLT2i and DPP4i is a suitable option for Indian T2DM patients that provides a safer, rapid, and sustained glycemic control, improves both insulin resistance and β-cell function, has extra-glycemic benefits that help reduce body weight and blood pressure, improves adherence and compliance by reducing the pill burden, and is cost-effective [6]. The DPP4i and SGLT2i have shown benefits in clinical trials and are recommended as add-on therapies for T2DM patients at risk or with CV or renal conditions that not only improve diabetes outcomes but also enhance CV and renal health [12].

Both SGLT2i and glucagon-like peptide-1 receptor agonists (GLP-1 RA) are the preferred therapies for T2DM and cardiorenal conditions. While both SGLT2i and GLP-1 RA demonstrate greater efficacy than DPP4i in lowering the risk of various cardiorenal outcomes, SGLT2i are particularly more effective than GLP-1 RA in decreasing the incidence of hospitalization for HF and renal events whereas GLP-1 RA mainly reduce the risk of nonfatal strokes [13]. Early introduction of SGLT2i within the first two years of diagnosis may help mitigate the legacy effect, offering long-term benefits for patients [12].

Capitalizing on the outcomes from numerous dedicated CV outcome trials (CVOTs), there is a wealth of information to guide the preferred selection of certain blood glucose-lowering drugs to lower CV risk [14]. Evidence suggests that a synergistic and rational combination of dapagliflozin, a SGLT2i, and sitagliptin, a DPP4i, is a suitable therapeutic option reported to have positive CV outcomes [15]. In the Dapagliflozin Effect on Cardiovascular Events-Thrombolysis in Myocardial Infarction 58 (DECLARE-TIMI 58) trial in patients with T2DM with or at risk for ASCVD, treatment with dapagliflozin showed no major adverse CV events (MACE) compared to placebo but led to decreased incidence of CV death or hospitalization for HF. There was a 17% reduction in CV death or hospitalization for HF with dapagliflozin (95% CI: 0.73-0.95, p = 0.005) [16]. There is strong clinical evidence for the use of FDC of dapagliflozin and sitagliptin in patients with uncontrolled T2DM, elderly T2DM patients, and T2DM patients with HF/high CV risk/established ASCVD/chronic kidney disease (CKD)/high risk of hypoglycemia [17].

To assess the therapeutic potential of the FDC of dapagliflozin and sitagliptin, two expert consensus meetings were previously convened. The initial meeting concentrated on the potential of the FDC of dapagliflozin and sitagliptin to meet unmet CV needs in people with T2DM and aimed to identify the most appropriate patient profiles for its application. The subsequent meeting addressed the underutilization of the FDC of dapagliflozin and sitagliptin in people with T2DM, despite existing guidelines advocating for the early initiation of dual therapy. The findings from the first meeting have been published, while the outcomes of the second are currently in the publication process. This expert consensus meet sought to understand the barriers to the practical implementation of the FDC of dapagliflozin and sitagliptin in practice and to strengthen evidence-based recommendations for its early initiation in people with T2DM and CV risk factors in the Indian context.

## Materials and methods

Seven advisory board meetings were conducted between May 11, 2024, and June 28, 2024, in various Tier 1 and Tier 2 cities across India. The goal was to discuss the barriers associated with the early initiation of FDC of SGLT2i, dapagliflozin, and DPP4i, sitagliptin, in managing people with T2DM at high CV risk established CVD and explore how this FDC could address the unmet CV needs of people with T2DM with high CV risk. These meetings engaged 98 cardiologists, including both academicians and clinicians with private practices, who were randomly selected from metropolitan cities across India. The meeting aimed to formulate expert opinion and evidence-based clinical guidance for appropriate consideration for combination therapy with SGLT2i + DPP4i and early initiation of FDC of dapagliflozin and sitagliptin in the routine clinical practice setting for the management of people with T2DM and CV risk. A comprehensive review of the available literature in PubMed and Google Scholar focusing on guidelines, meta-analyses, systematic reviews, randomized controlled trials (RCTs), non-RCTs, real-world studies, and key articles related to SGLT2i and DPP4i as drug classes, their combination therapies, and the FDC of dapagliflozin and sitagliptin was done to provide evidence-based, practical recommendations for the optimal clinical application of this combination to enhance glycemic control and reduce CV events in people with T2DM and multiple CV risk factors. Discussions during the meetings were structured around a questionnaire aimed at understanding cardiologists' perceptions and pharmacotherapeutic strategies on the use of SGLT2i and DPP4i as a drug class, and FDC of dapagliflozin and sitagliptin in patients with T2DM and multiple CV risk factors or established CVD. The questionnaire was reviewed and validated by 10 key opinion leaders with over 20 years of experience in the field. Polling was conducted through the Mentimeter app (Stockholm, Sweden) to collect structured responses. All experts participating in the advisory meetings were asked to provide answers based on their clinical practice experience. Opinions were collected using a cross-sectional method, and consensus was achieved by equal participation and shared discussion among the experts and voting.

## Results

Comprehensive management of CV risk factors for adults with T2DM

People with diabetes are two to four times more likely to develop CVD than those without diabetes. Coronary artery disease, HF, and stroke are the major causes of death and disability in people with diabetes [18]. A considerable number of patients with CVD have undetected T2DM. Diabetes with cardiorenal co-morbidities increases the likelihood of having CV events and all-cause mortality. People diagnosed with diabetes should be evaluated for the existence of ASCVD and target organ damage that can help in treatment decisions to reduce the risk of CVD [14]. Treatment optimization, including careful selection of pharmacotherapy with particular attention to CV safety, is important in diabetes patients [19].

Expert Opinion

CV risk reduction should be prioritized in all people with T2DM, as it is essential for effectively managing their overall health. The primary goal of pharmacotherapy in people with T2DM is to reduce the HbA1c levels and minimize the risk of future CV events.

When choosing an antidiabetic drug for people with T2DM, one needs to take into account the patient's age, severity of diabetes, co-existing medical conditions, susceptibility to hypoglycemia, tolerance to specific drugs, and concerns about weight gain. The antidiabetic medication selected should help not only manage blood glucose levels effectively but also have beneficial effects on the end organs without causing any harmful effects for people with T2DM with CV risks or established CVD.

Early intensive glycemic control with combination therapy

A one-year delay in treatment intensification combined with inadequate glycemic control significantly raises the risk of myocardial infarction (MI), HF, stroke, and composite CV events [20]. The risk of diabetes complications is increased even with brief episodes (weeks) of hyperglycemia, which emphasizes the importance of achieving optimal glycemic control as early as possible [21].

**Figure 1:**
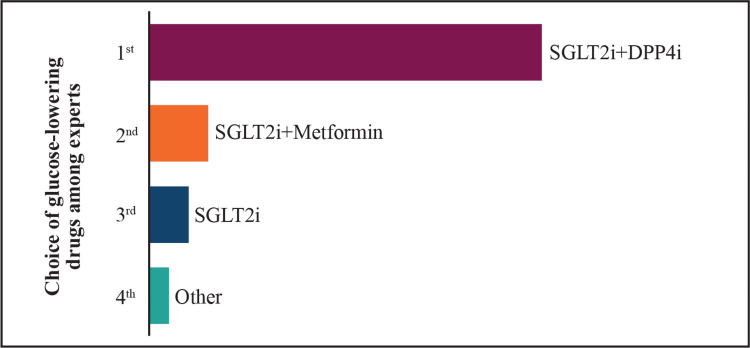
Preference for glucose-lowering drugs in T2DM patients with ASCVD risks (results of the expert poll conducted on the Mentimeter app) SGLT2i: sodium-glucose co-transporter-2 inhibitors; DPP4i: dipeptidyl peptidase-4 inhibitors; T2DM: type 2 diabetes mellitus; ASCVD: atherosclerotic cardiovascular disease

Recommendations for selecting the most appropriate glucose-lowering drug for the treatment of T2DM have changed over the last decade based on the evolving evidence (Figure 1). The 2018 American Diabetes Association (ADA)/European Association for the Study of Diabetes (EASD) consensus showed a paradigm shift from a treat-to-goal approach to a treat-to-benefit approach, which was driven by the results from CVOTs that demonstrated the benefits of SGLT2i and GLP-1 RAs on hypoglycemic risk, CV outcomes and extra-glycemic benefits on weight, and blood pressure [22].

Expert views on therapy preference in people with T2DM with ASCVD risks

The definition of intense therapy has changed in the recent decade with the availability of new antihyperglycemic medications that have a lower risk of hypoglycemia in comparison to sulfonylureas and insulin and provide additional alternatives for combination therapy targeted at improving glycemic control [21].

Combination therapy may be the optimal strategy for people with T2DM with an HbA1c of >8.0% at initial diagnosis. In most clinical trials, initial dual combination therapy has demonstrated greater reductions in HbA1c and a greater likelihood of achieving target HbA1c compared to monotherapy. The reduction in HbA1c with initial dual therapy is about 1%-2%, depending on the type of drug and the baseline HbA1c levels [23].

Expert Opinion

Strict glycemic control is crucial not only for improving blood glucose levels but also for target organ protection in people with T2DM. Even a modest reduction of 1% in HbA1c can significantly impact CV events and reduce mortality rates. Majority of the cardiologists strongly agreed that monotherapy alone is not adequate for managing people with T2DM with CVD risk.

The optimal approach for managing people with T2DM at risk of complications is shifting from the stepwise "treat-to-failure" approach to a "treat-to-target" approach with an aim to maintain HbA1c levels below 7% or ideally below 6.5%. The treatment should be intensive and aggressive, with combination therapy to achieve glycemic control and also prevent long-term complications. Patients with high HbA1c levels at diagnosis or those who do not reach the target HbA1c within three months should be started on combination therapy, as a delay beyond three months is not recommended. Patients with an HbA1c of 8% or higher at diagnosis should begin combination therapy immediately. Also, patients with an HbA1c of 1.5%-2% above the target should be started on a dual-drug combination, which can later be de-escalated once glycemic control is achieved.

Barriers to achieving glycemic control in T2DM patients

As per the Indian Council of Medical Research-India Diabetes (ICMR-INDIAB) study, about 69% of people with T2DM have suboptimal glycemic control [6]. A significant proportion of people with T2DM are at risk of having inadequate glycemic control for years before receiving more intensive treatment due to clinical inertia, which can be due to various reasons relating to physicians, patients, and the healthcare system [24,25].

This may cause T2DM patients to live with inadequate glycemic control for many years, which can have an impact on their quality of life and increase the risk of CV events, thereby increasing mortality and morbidity and eventually increasing healthcare resource utilization and cost [26,27].

**Figure 2:**
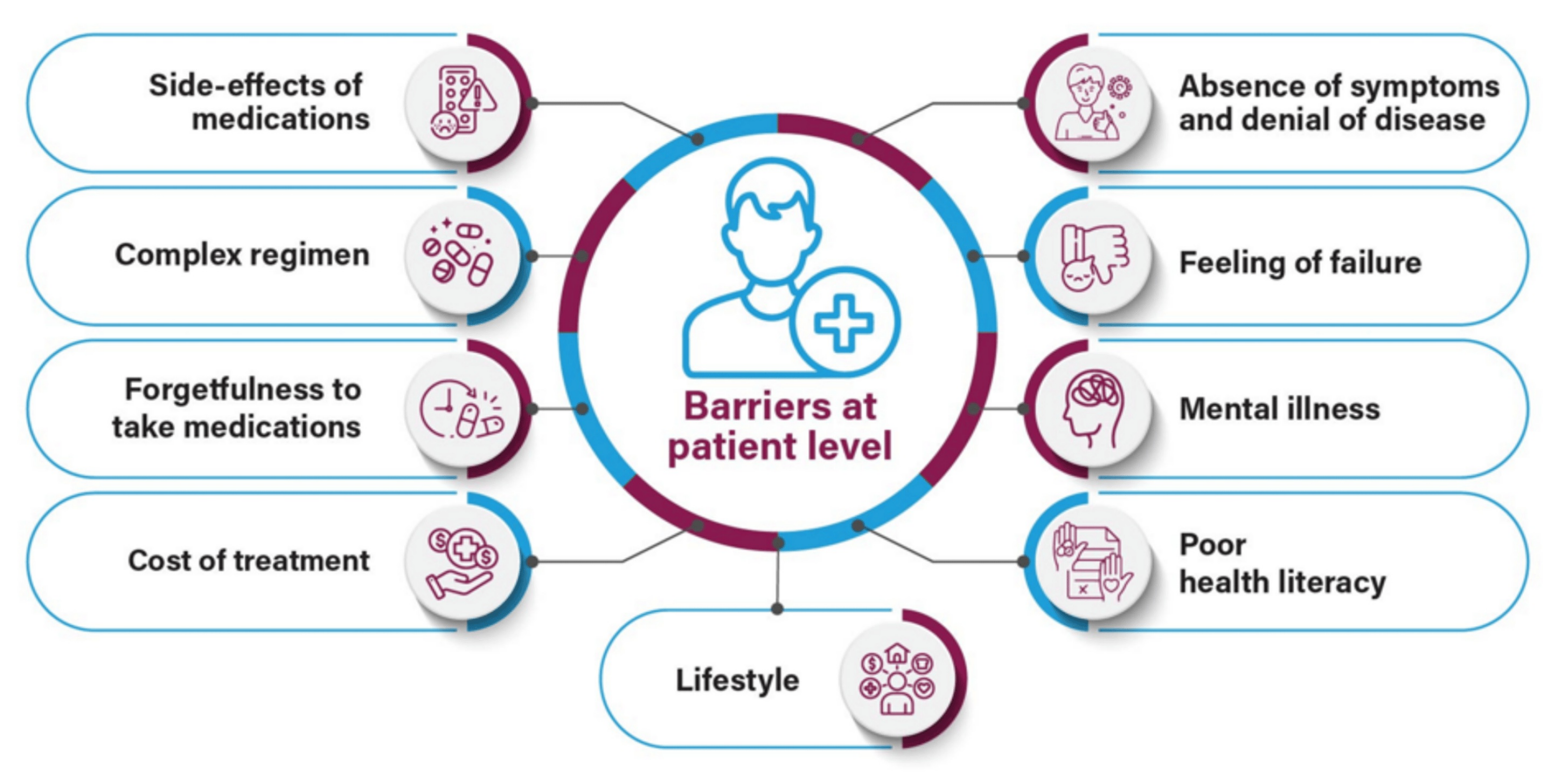
Barriers at the patient level Image has been created by the authors using information from Ross [24].

Nonadherence to therapy is one of the major barriers to optimum glycemic control in patients with T2DM, which can lead to poor treatment outcomes and increased utilization of healthcare resources. Patients may be reluctant to initiate or intensify treatment due to feelings of failure about suboptimal glycemic control, anxiety about hypoglycemia and weight gain, fear of injections, complexity of disease management, inconvenience, and poor education about T2DM and the available therapies (Figure 2) [24].

**Figure 3:**
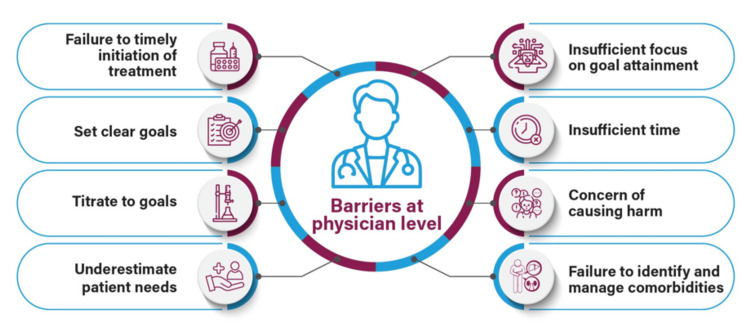
Barriers at the physician level Image has been created by the authors using information from Ross [24].

The physician-level barriers to optimal glycemic control are often referred to as clinical inertia or benign neglect that can be due to numerous factors, including time and resource constraints, overly cautious prescribing practices to avoid side effects, underestimation of patient needs, failure to set clear treatment goals, or lack of encouragement to reach goals (Figure 3) [24].

**Figure 4:**
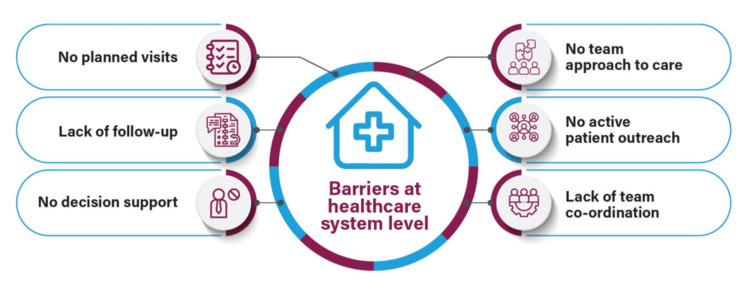
Barriers at the healthcare system level Image has been created by the authors using information from Ross [24].

In addition to physician-level barriers, shortcomings within the healthcare system (Figure 4) can also play a role in patients' poor adherence to treatment or hesitancy to intensify their therapy [24].

**Figure 5:**
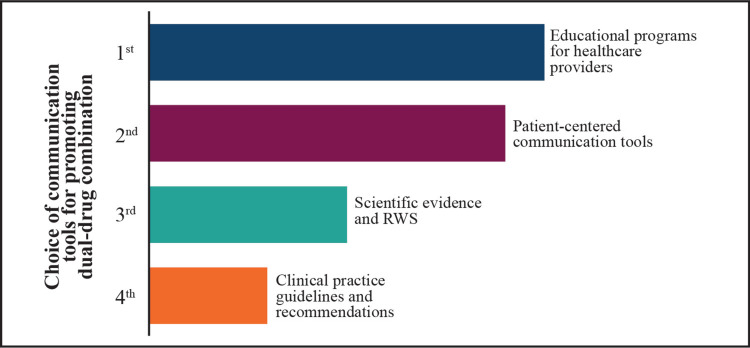
Tools for promoting combination therapy in practice (results of the expert poll conducted on the Mentimeter app) RWS: real-world studies Image credits: the authors

Inadequate glucose control, as well as challenges that affect patients' adherence to diabetes treatment plans, needs to be reinforced with timely follow-up and escalation or intensification of treatment as needed. It is important to provide proper patient education on the progressive nature of T2DM, the risks associated with long-term poor glycemic control, and the need to adhere to the therapeutic regimens (Figure 5) [27-29].

Expert views on communication for the initiation of combination therapy in people with T2DM 

Expert Opinion

One barrier to good glycemic control in people with T2DM is the treat-to-failure approach. Misunderstandings about diabetes are also a barrier to achieving good glycemic control. Issues like medication accessibility, availability, and cost can lead to low patient adherence. Hesitancy to change prescriptions from referral physicians (diabetologists/endocrinologists/consulting physicians) is also one of the barriers to good glycemic control.

Complex pathophysiology is also a challenge that makes it difficult to actually decide which combination therapy. Clinical inertia due to patient resistance to treatment modifications, pill burden, and concerns of side effects are significant barriers to the early initiation of dual-drug FDC in people with T2DM with CV risk. Also, the risk of complications such as hypoglycemia, weight loss (as some cardiac patients post-coronary artery bypass grafting (CABG) or post-angioplasty already have significant weight loss), and infections (urinary tract or genital mycotic infections) can deter treatment changes. Also, failure to initiate combination therapy early, despite guideline recommendations, is a significant barrier.

Overcoming these barriers requires strategies like interactive workshops, research participation, patient-centered education, peer-to-peer education, and collaboration across specialties.

The legacy effect of early tight glycemic control

The legacy effect begins as early as the first year after diagnosis and depends on the level of glycemic exposure. In comparison to HbA1c levels < 6.5% for the first year after diagnosis, levels ≥ 6.5% in the first year after diagnosis are linked to an increased risk of future diabetic complications. The HbA1c levels of ≥7% in the first year after diagnosis are associated with an increased risk of future mortality, and HbA1c levels ≥ 8% are associated with an increased risk of microvascular complications and mortality. Therefore, it is essential to achieve glycemic control soon after diabetes diagnosis to avoid the sequelae of diabetic complications [30].

Patients subjected to early intensive glycemic control have a legacy benefit that lasts 10 and 30 additional years, as reported in the United Kingdom Prospective Diabetes Study (UKPDS) and the Diabetes Chronic Complications Trial (DCCT) study, respectively [31,32].

The availability of newer agents that offer CV protection without increasing hypoglycemia risk enhances the options for safely achieving glycemic targets. An early, aggressive, and multifaceted approach to T2DM management establishes optimal metabolic memory and is the best strategy. Personalized use of newer CV-superior therapies, alongside conventional treatments like metformin and sulfonylureas, is recommended to optimize outcomes. Early, intensive glycemic control offers long-term benefits in terms of reducing complications and improving survival, establishing it as the standard approach for treating newly diagnosed T2DM [33].

Poor glycemic control during the first three years after diabetes diagnosis increases the risk of future CVD. The SGLT2i initiated within the first two years can attenuate the phenomenon of the legacy effect. Early treatment with SGLT2i may, therefore, provide lasting benefits for patients who fail to achieve optimal glycemic control after T2DM diagnosis. In newly diagnosed persons with T2DM with no CVD at baseline, a mean HbA1c level ≥ 7% or ≥8% during 0-3 years after diagnosis is associated with a higher risk of subsequent CVD compared to HbA1c levels ≤ 7%. These associations are not seen when SGLT2i are introduced in the first two years of diagnosis [34]. The robust evidence supporting the benefits of SGLT2i in reducing or preventing serious CV, renal, and metabolic complications, while ensuring long-term health outcomes, should outweigh manageable concerns, such as mycotic genital infections, and the perceived risk of rare adverse events that may have previously limited their prescription to eligible individuals [35].

Early intensive and sustained glycemic control has potential long-term benefits. Early treatment intensification with DPP4i within two years of diabetes diagnosis reduces glycemic variability, improves glycemic durability, and delays insulin initiation [36].

Achieving an HbA1c of <7.5% within the first year after a T2DM diagnosis is associated with a reduced risk of MACE, and maintaining minimal variability thereafter further lowers the risk of MACE [37]. Early combination therapy improves glycemic profiles without increasing the frequency of adverse effects and has a positive legacy effect [38]. There is strong evidence to suggest the legacy effect following intensive intervention on CV risk factors, though its impact varies by risk factor, patient characteristics, disease duration, and type of intervention. The legacy effect seems to be pronounced in those with moderately high CV risk but no CVD, particularly in those with recent-onset diabetes [39]. Better medication adherence is also associated with a lower risk of cardiocerebrovascular events and all-cause mortality [40].

Expert Opinion

The longer the patient is not at glycemic goal, the more detrimental it is to the vasculature. Strict glycemic control not only improves blood glucose levels but also offers target organ protection. Intensive glycemic control soon after a diabetes diagnosis can help reduce complications and lower mortality by up to 10 years or longer. Achieving good glycemic control within the first four to five years following diabetes diagnosis can reduce microvascular and macrovascular complications of diabetes over the long term.

Combination therapy for glycemic control should be initiated, as recommended by the guidelines. Early intensive glycemic control with combination therapy is recommended to reach the HbA1c goal. Combination therapy with metformin helps achieve the target HbA1c more rapidly than monotherapy. Introducing SGLT2i early, within two years of diagnosis, can help ameliorate the problem associated with sustained high blood glucose levels.

Merits of choosing FDC

**Figure 6:**
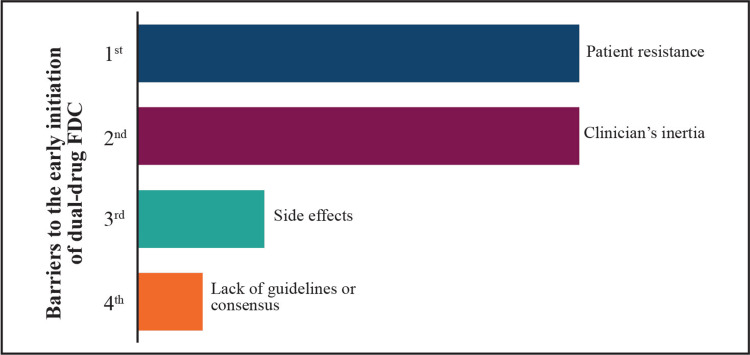
Major barriers to the early initiation of dual-drug FDC in T2DM patients with CV risk (results of the expert poll conducted on the Mentimeter app) FDC: fixed-dose combination; T2DM: type 2 diabetes mellitus; CV: cardiovascular Image credits: the authors

The first-line drug often falls short of achieving adequate glycemic control due to the progressive nature of T2DM, thereby requiring add-on therapy [38]. Multiple patient- and clinician-level barriers (Figure 6) lead to clinical inertia with glycemic targets not being reached and poor outcomes as a consequence. This may be partly due to clinicians not managing patients optimally or in accordance with guidelines [41].

Expert views on barriers to initiation of dual-drug FDC

The main cause of uncontrolled glycemia (not at target HbA1c) is patients' resistance to increasing the pill burden. This can be due to concerns about the added expense, potential inconvenience, and possible negative effects of increased pill burden [38].

Suboptimal adherence to pharmacotherapy in patients with T2DM and CVD is partly due to pill burden, where the FDCs are one way of reducing the pill burden in those who require multiple medications [42]. FDCs can help achieve glycemic targets effectively with an advantage over individual therapy, such as increased patient adherence due to reduced complexity of dosing, greater efficiency, and cost-benefit. The rationale for choosing FDCs is that the drugs in combination should have different mechanisms of action, their pharmacokinetics should not differ too greatly, the combination should not have additive effects that induce supra-additive toxicity, and they should be selected based on guideline recommendations, as to whether they support its use or raise any concerns [38].

The FDC and fixed-ratio combination (FRC) therapies with complementary mechanisms offer the potential for earlier, more sustainable glycemic control, improved patient adherence, and reduced side effects and may also help slow disease progression, lower the risk of vascular complications, and provide cost savings compared to the use of individual medications [43].

The advantages of FDCs include reducing the medication burden on patients, improving adherence rates, enhancing glycemic control with better efficacy, and being cost-effective. They also reduce the frequency of drug administration and help prevent polypharmacy. However, the disadvantages include challenges with dose titration, difficulty in switching patients who are satisfied with separate medications, a potential increase in adverse drug reactions, and possible effects on the bioavailability of the agents. A systematic review of FDCs of oral hypoglycemic agents shows they effectively control hyperglycemia in patients with T2DM and help achieve target glycemic levels, which also reduces long-term complications and improves patients' quality of life [44].

Prescribing DPP4i in FDCs is considered a positive prescribing choice to enhance compliance and leads to improved effectiveness of glucose-lowering drugs in the real world. From the patient’s perspective, the decision to prescribe an FDC is associated with better medication adherence and treatment satisfaction, which are consistent with the results of a systematic literature review [45].

Expert Opinion

The choice of antidiabetic drug combinations should be individualized to prioritize comprehensive patient-centered cardiometabolic risk management. Pill burden should be reduced to improve treatment adherence in people with T2DM with ASCVD risk or CVD. The FDC may be chosen for simpler dosing, reduced pill burden, and lower risk of medication error and to improve adherence, which ultimately leads to better treatment outcomes.

Synergistic effects of the combination of SGLT2i + DPP4i in the management of T2DM

The combined usage of SGLT2i and DPP4i either separately or in FDC helps address at least six of the eight elements in the "ominous octet." They offer "treat-to-target" benefits either after metformin initiation or before metformin initiation (metformin is contraindicated or intolerant) or in patients with high HbA1c who fail on metformin treatment, which supports the "treat-early-and-treat-right" approach [6,15].

The combination of a SGLT2i and a DPP4i is an attractive approach for managing T2DM. Combination therapy is more efficacious than monotherapy in controlling blood glucose without compromising safety. The two drugs work through different complementary mechanisms: DPP4i provides a strong safety profile with no increased risk of hypoglycemia, weight gain, or CV events, while SGLT2i targets the kidneys to promote glucosuria. In addition to lowering glucose, SGLT2i contributes benefits such as weight reduction (including abdominal fat), lower blood pressure, and decreased serum uric acid, all of which are independent CV risk factors [46].

Combination therapy with SGLT2i and DPP4i results in a greater reduction in HbA1c (weighted mean difference (WMD) -0.6%, 95% CI: -0.7 to -0.5%), fasting plasma glucose, two-hour PPBG, and body weight compared to placebo and DPP4i. Both simultaneous combination therapy and sequential addition of SGLT2i to DPP4i lead to more significant reductions in HbA1c (WMD -0.49%, 95% CI: -0.61 to -0.38%, and WMD -0.65%, 95% CI: -0.78 to -0.52%, respectively) than with placebo/DPP4i. While there was a trend toward a greater HbA1c reduction with the sequential addition of SGLT2i to DPP4i compared to the simultaneous combination, this difference was not statistically significant. The risk of hypoglycemia was higher with the SGLT2i/DPP4i combination only when insulin or sulfonylureas were used as background therapy. Although the risk of UTIs did not increase with SGLT2i/DPP4i, the risk of genital infections was higher when SGLT2i was added to an existing DPP4i regimen [11]. The risk of genital tract infections (GTIs) is lower and the incidence of UTIs is reduced nominally with simultaneous combination as opposed to sequential combination of SGLT2i and DPP4i. The risk of UTI is found to be slightly higher in those receiving sequential combination therapy (relative risk (RR): 0.96, 95% CI: 0.52-1.78) vs. simultaneous combination group (RR: 0.67, 95% CI: 0.28-1.60), and the risk of a GTI was also higher in the sequential combination group (RR: 5.57, 95% CI: 2.33-13.33) vs. simultaneous combination (RR: 1.35, 95% CI: 0.55-3.34) [6].

In a large comparative effectiveness and safety study involving 144,614 adults with T2DM, treatment initiation with either SGLT2i (n = 60,523) or a DPP4i (n = 84,091) showed that SGLT2i benefits patients regardless of their glycemic control. The adverse effect profile of SGLT2i is similar to that of DPP4i, with no increased risk of adverse effects in patients with elevated HbA1c levels [47].

**Figure 7:**
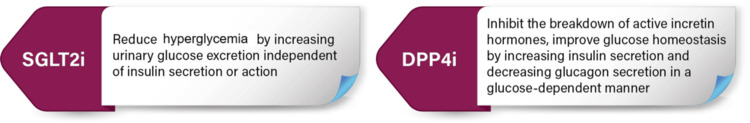
Complementary effects of SGLT2i and DPP4i SGLT2i: sodium-glucose co-transporter-2 inhibitors; DPP4i: dipeptidyl peptidase-4 inhibitors Image has been created by the authors using information from Cho et al. [10].

The combination of SGLT2i and DPP4i in FDC offers several advantages. It provides safer, rapid, and sustained glycemic control, with improvement in insulin resistance and β-cell function. In addition to glycemic benefits, it also offers extra-glycemic benefits including reductions in body weight and blood pressure. It enhances patient adherence and compliance, by reducing the pill burden, making treatment more manageable, and is also cost-effective with clinical and economic benefits [6]. The SGLT2i and DPP4i combination with complementary mechanisms of action may enhance the glucose-lowering effect (Figure 7) without compromising drug safety and rarely cause common adverse effects of other oral antidiabetic drugs, such as weight gain and hypoglycemia [11].

**Figure 8:**
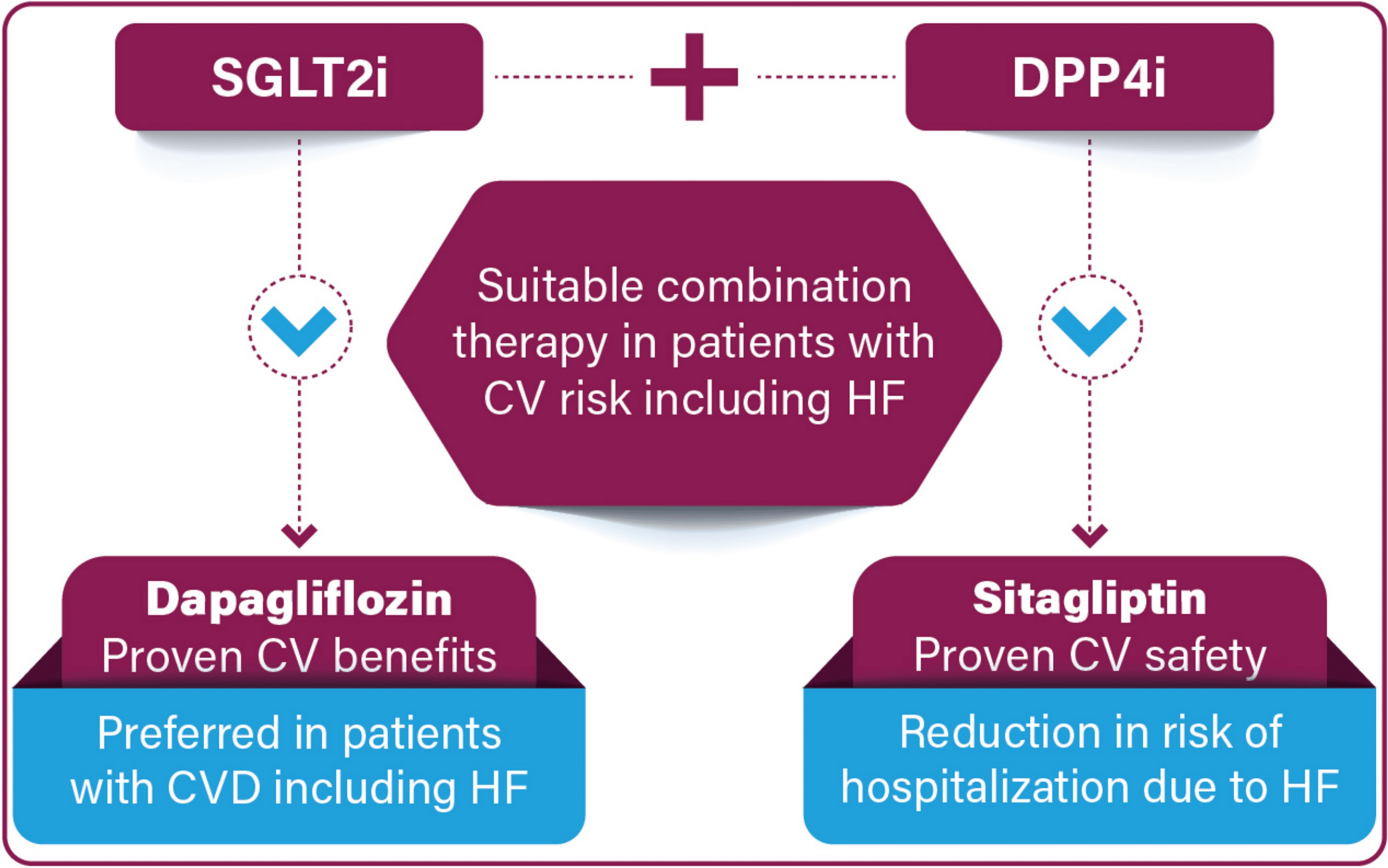
SGLT2i and DPP4i in T2DM with CV risk and/or HF risk SGLT2i: sodium-glucose co-transporter-2 inhibitors; DPP4i: dipeptidyl peptidase 4 inhibitors; CVD: cardiovascular disease; T2DM: type 2 diabetes mellitus; CV: cardiovascular; HF: heart failure Image has been created by the authors using information from Saikia et al. [12].

Many current guidelines and expert opinions recommend the use of SGLT2i early in the management of T2DM due to their proven positive effects in reducing and/or preventing diabetes-related CV and metabolic complications and clinical and economic benefits [35]. The combination of SGLT2i with DPP4i has been shown to achieve glycemic control versus monotherapy in cases with uncontrolled hyperglycemia. Also, the combination of SGLT2i with DPP4i that targets several pathophysiological pathways is reported to have certain CV benefits (Figure 8) [15].

**Figure 9:**
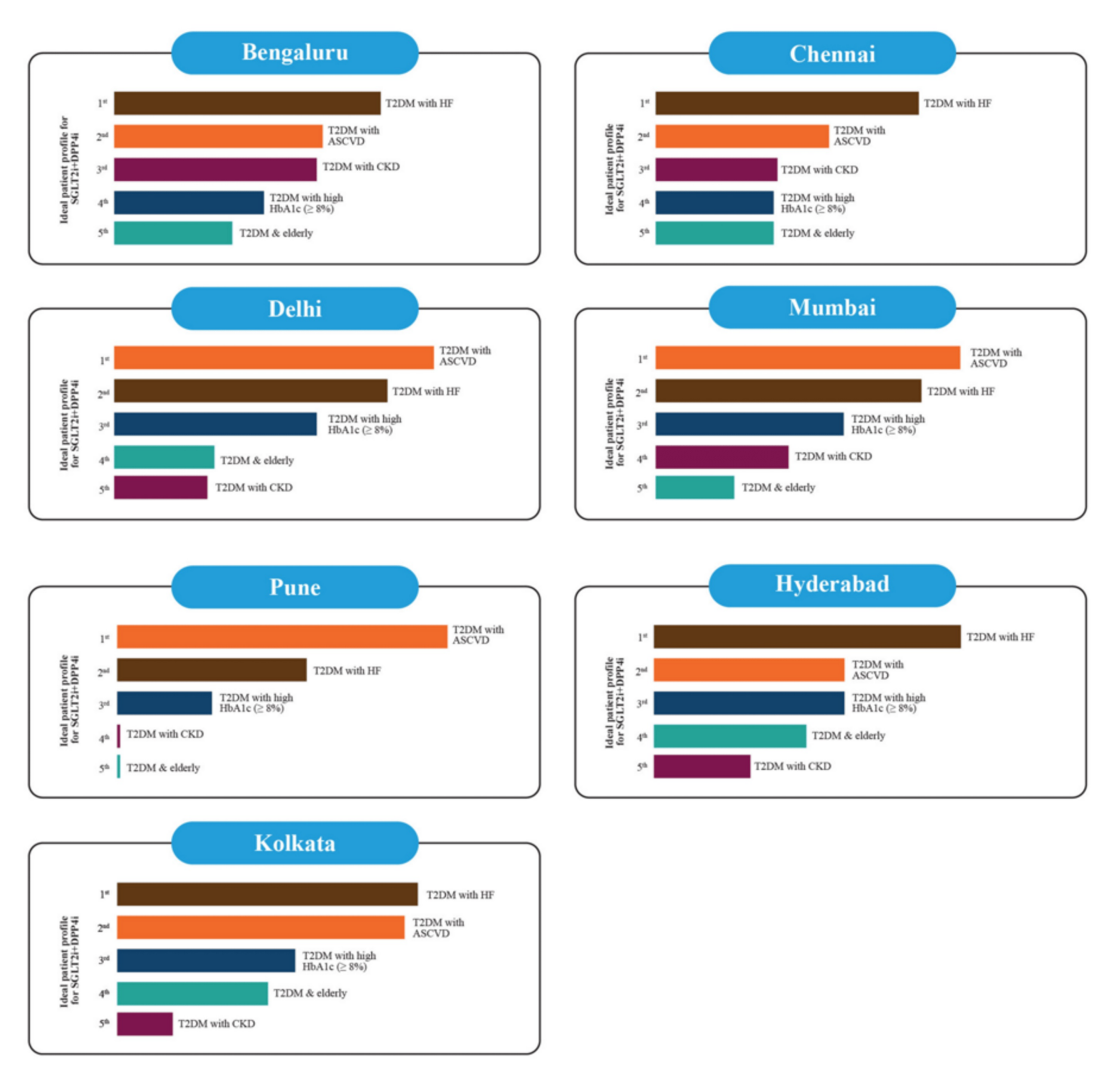
Ideal patient profile for prescribing the SGLT2i+DPP4i (results of the expert poll conducted on the Mentimeter app) SGLT2i: sodium-glucose co-transporter-2 inhibitors; DPP4i: dipeptidyl peptidase 4 inhibitors; ASCVD: atherosclerotic cardiovascular disease; T2DM: type 2 diabetes mellitus; HF: heart failure; HbA1c: glycated hemoglobin; CKD: chronic kidney disease Image credits: the authors

The FDC of SGLT2i and DPP4i provides glycemic and pleiotropic effects, including a lower risk of hypoglycemia, lower rates of genitourinary tract infections, and weight neutrality. The combination is preferred (Figure 9) over other conventional therapies with no CV benefit in cases of established CVD and/or HF risk [17].

The overall safety profile of the FDC of SGLT2i and DPP4i is similar to that of individual components with no significant differences in hypoglycemia events, UTIs, or events related to hypovolemia and ketoacidosis. However, the rates of GTIs reported are slightly lower with the FDC as compared to SGLT2i monotherapy, and the probable reasons for such moderation of GTIs with the FDC, beyond improved glycemic control, may be the interaction of DPP4 and SGLT2 proteins at the renal tubular cell-membrane level, or the inhibition of the DPP4 enzyme present in certain pathogenic microbes that may render them inactive [6].

Expert views on the ideal patient profile for prescribing the SGLT2i + DPP4i

The combination of SGLT2i and DPP4i is more relevant in patients with high CV risk and/or HF risk who have HbA1c > 1.5% above the individualized target [6]. The SGLT2i strongly bind to plasma proteins, while DPP4i do not. Although a theoretical interaction can exist between a DPP4i and a SGLT2i due to shared transporters, studies on drug interactions have not shown significant pharmacokinetic changes. A clinically significant interaction is unlikely given the safety margins of both drug classes and the available interaction data. Co-administering a DPP4i and a SGLT2i is a promising treatment option as their complementary mechanisms of action do not affect the safety profile of either agent [48].

The use of a DPP4i and SGLT2i combination as an early add-on strategy is crucial for achieving and maintaining treatment goals in patients with the Asian-Indian T2DM phenotype. This combination addresses key T2DM-related pathophysiological issues, such as β-cell dysfunction, hepatic insulin resistance, and hyperglucagonemia, while also offering extra-glycemic benefits, including improvements in weight, blood pressure, and cholesterol levels that contribute to better outcomes for Indian diabetic patients with CV risk or metabolic traits [49].

Expert Opinion

Combination therapy with complementary mechanisms of action is preferred to address the multiple pathophysiologic abnormalities in people with T2DM. Most cardiologists agreed that the FDC combination of SGLT2i and DPP4i is essential for addressing CV co-morbidities with T2DM. The most compelling benefit of FDC of SGLT2i and DPP4i is improved glycemic control with CV risk reduction.

SGLT2i should be used cautiously in patients with a history of CABG or angioplasty, particularly those with significant weight loss or urinary/genital fungal infections. Elderly patients and individuals with CKD should be carefully assessed on a case-by-case basis before initiating the FDC of SGLT2i and DPP4i.

Rationale for the early initiation of FDC of dapagliflozin plus sitagliptin

Early intensive therapeutic management of T2DM is beneficial for clinical outcomes where a dual combination therapy of dapagliflozin and sitagliptin may be an important part of the armamentarium available to adequately manage cardiometabolic risk in T2DM. In contrast to various other oral antidiabetic medications, the action of dapagliflozin is independent of insulin secretion and action [15].

Dapagliflozin, a highly selective SGLT2i, limits the reabsorption of filtered glucose in the kidney, increasing urine glucose excretion and reducing blood glucose levels. It has a mode of action that is independent of pancreatic β-cell function and regulation of insulin sensitivity [50,51]. Dapagliflozin is a highly potent (inhibitory constant 0.55 nmol/L) and reversible SGLT2i, which is >1,400 times more selective for SGLT2 than SGLT1, the main transporter responsible for glucose absorption in the gut [52].

The DPP4i, when used in combination with other antidiabetic medications, provides a complementary effect via its unique mechanism of action. The mechanism of action of sitagliptin is to competitively inhibit the DPP4 enzyme, which breaks down the incretins, GLP-1 and gastric inhibitory polypeptide (GIP), and gastrointestinal hormones released in response to a meal. By preventing the breakdown of GLP-1 and GIP, they increase the secretion of insulin and suppress the release of glucagon by the alpha cells of the pancreas. This drives the blood glucose levels toward normal, and as the level approaches normal, the amounts of insulin released and glucagon suppressed diminishes. This tends to prevent an "overshoot" and subsequent hypoglycemia, which is seen with some other oral hypoglycemic drugs [15].

Positive CV outcomes have been reported for SGLT2i and DPP4i, particularly with the combination therapy of dapagliflozin and sitagliptin, which seems to be a suitable therapeutic option. The broad range of clinical outcomes associated with this combination, including improved glycemia and obesity, reduced metabolic and vascular risk, and safety, are highly relevant to the Asian-Indian phenotype of T2DM [15].

A randomized trial evaluated the efficacy and safety of dapagliflozin 10 mg (n = 225) versus placebo (n = 226) as add-on therapy to sitagliptin 100 mg, with or without metformin, in patients with inadequately controlled T2DM (mean baseline HbA1c 7.9%). After 24 weeks, dapagliflozin resulted in significant clinical benefits, including a reduction in HbA1c (-0.5% vs. 0% with placebo) and body weight (-2.1 kg vs. -0.3 kg). These improvements in glycemic control and weight were sustained through week 48 [53].

The FDC of sitagliptin and dapagliflozin significantly improves glycemic control and lipid profiles in T2DM patients, especially those with coronary artery disease. It has a favorable safety profile in the Indian population, highlighting its potential as an effective and well-tolerated treatment option for patients with established CVD [54]. Compared to sitagliptin, dapagliflozin is found to be more effective at improving cardiometabolic risk factors. Dapagliflozin was superior in reducing fasting plasma glucose, insulin, and uric acid, increasing high-density lipoprotein cholesterol, and suppressing the increase in serum creatinine and the decrease in estimated glomerular filtration rate. On the other hand, sitagliptin was superior to dapagliflozin in suppressing glucose variability [55]. In patients aged ≥65 years with T2DM and mild renal insufficiency who had inadequate glycemic control on metformin ± sulfonylurea, treatment with sitagliptin for 24 weeks led to an improvement in HbA1c, relative to treatment with dapagliflozin. Both sitagliptin and dapagliflozin were generally well tolerated [56].

Triple FDC of dapagliflozin + sitagliptin + metformin extended-release (ER) tablets once daily was significantly better in achieving glycemic control vs. dual-combination once daily in patients with T2DM poorly controlled with metformin without any significant safety concerns. At week 16, adjusted mean reduction in HbA1c from baseline was significantly greater with dapagliflozin + sitagliptin + metformin ER (-1.73%) compared to sitagliptin + metformin sustained-release (SR) (-1.28%, difference of -0.46%, p < 0.001) and dapagliflozin + metformin ER (-1.33%; difference of -0.4%, p < 0.001). At week 16, dapagliflozin + sitagliptin + metformin ER showed a significant reduction in PPBG compared to dapagliflozin + metformin ER (p = 0.0394) and a significant reduction in fasting blood glucose with dapagliflozin + sitagliptin + metformin ER compared to sitagliptin + metformin SR (p = 0.0226). The proportion of patients achieving HbA1c < 7% at week 16 was significantly higher with dapagliflozin + sitagliptin + metformin ER (38.5%) vs. sitagliptin + metformin SR (12.8%) (p < 0.001) and dapagliflozin + metformin ER (21.3%) (p = 0.0023) [57].

Expert Opinion

CV risk reduction should be addressed simultaneously in all patients with T2DM with the goal of reducing future CV events. As CV risk is a significant concern in T2DM patients, it is necessary to initiate SGLT2i in all T2DM patients with co-morbid conditions to mitigate the risk of HF. Dapagliflozin is more potent among SGLT2i, with wider acceptability and evidence base.

DPP4i may be considered for achieving reduced HbA1c levels. The most commonly prescribed are sitagliptin, linagliptin, and vildagliptin, with linagliptin being a preferred option for patients with renal impairment. Sitagliptin has comparable efficacy and CV safety to other DPP4i, except saxagliptin. Sitagliptin has cardioprotective effects in diabetic patients with acute coronary syndrome and patients at high risk of HF after acute coronary syndrome, with no increased risk of de novo HF or other adverse CV events.

A combination of dapagliflozin and sitagliptin is recommended for people with T2DM with CV risk and may be prescribed at diagnosis in people with T2DM with high CVD risk. The rationale for the early initiation of FDC of dapagliflozin plus sitagliptin is its potential synergistic effects with rapid and sustained glycemic control, early achievement of HbA1c target, improvement of insulin resistance and obesity, reduction in metabolic and vascular risk, and lower risk of hypoglycemia. The combined effect of dapagliflozin and sitagliptin in FDC provides rapid, safe, and sustained glycemic control in patients whose blood sugar cannot be controlled with metformin, with a significant reduction in HbA1c levels, change in body weight, and CV mortality benefit.

FDC of dapagliflozin and sitagliptin is an important part of the armamentarium available to adequately manage cardiometabolic risk in people with T2DM (Figure 8). It is more effective in treating diabetes with or without co-morbidities in the Asian-Indian phenotype. It has the advantages of once-daily dosing, a low risk of hypoglycemia, and CV and renal safety and is easily accessible and affordable.

The patient profile for FDC dapagliflozin and sitagliptin includes T2DM with HbA1c ≥ 8%, T2DM with HF, T2DM with ASCVD, T2DM with CKD, T2DM in the elderly (prevents hypoglycemia), T2DM and overweight, and T2DM uncontrolled on metformin.

## Discussion

Diabetes is not only associated with hyperglycemia but also leads to several long-term complications that can have serious implications on the health and well-being of those affected [18]. The goals of treatment in T2DM are to prevent or delay chronic consequences and preserve the patient's quality of life, which requires glycemic control and management of CV risk factors [58]. It is important to recognize and address the risk factors and co-morbidities as early as possible while managing T2DM with a multifactorial strategy and clear treatment goals [1,14].

CVD and diabetes commonly share risk factors and often require management that considers the presence or absence of the other [59]. The risk of CV events can be significantly reduced by implementing evidence-based therapies for the control or modification of multiple cardiometabolic risks in people with T2DM [1]. In individuals with T2DM, the CV risks are not sufficiently optimized by treatment approaches that solely target glucose control. Comprehensive and advanced strategies are needed to address CV risk factors in order to reduce the morbidity and mortality associated with CVD. The novel hypoglycemic drugs that are available improve CV outcomes independently and hold significant promise for revolutionizing the treatment of T2DM and associated CVD risk factors [60].

Initial HbA1c is the most important consideration when deciding whether to administer initial combination therapy to T2DM patients [23]. Various guidelines recommend early combination therapy in T2DM. The ADA guidelines recommend early combination therapy for individuals at treatment initiation to extend the time to treatment failure. They recommend considering initial combination therapy in people presenting with HbA1c levels of 1.5%-2.0% above target [7]. The American Association of Clinical Endocrinology (AACE) guideline recommends early combination therapy for those individuals recently diagnosed with T2DM with HbA1c ≥ 7.5% [8]. The Research Society for Study of Diabetes in India (RSSDI) guideline recommends considering initiating combination therapy if the HbA1c level is >1.5% above the target [9].

An early, proactive, and aggressive approach to treatment with combination therapy can improve glycemic profiles and have a positive legacy effect, as a longer time to transition from monotherapy to combination therapy and a longer time to treatment intensification can increase the risk of microvascular and macrovascular complications [38]. Although a pathophysiological approach using initial combination therapy with drugs known to address the established defects in T2DM seems more rational, it is preferable to use combination therapies with complementary mechanisms of action to target different pathways to address the multiple pathophysiologic abnormalities of T2DM [6].

Cardiometabolic risk is a distinctive feature of the Asian-Indian phenotype of T2DM. The selection of different antidiabetic drug combinations should be personalized within the framework of prioritizing patient-centric comprehensive cardiometabolic risk management [15]. Addressing multiple pathophysiological aspects of T2DM is crucial, and considering a combination therapy of SGLT2i and DPP4i may be pertinent in this context. A combination of DPP4i and SGLT2i might be a suitable option to consider along with metformin for the effective management of blood glucose levels to improve metabolic parameters without increasing the risk of hypoglycemia [6].

SGLT2i have demonstrated improved CV outcomes regardless of glycemia control and are now an essential part of the armamentarium for the management of cardiometabolic risk in T2DM patients [15]. The SGLT2i offer multiple nonglycemic benefits in people with T2DM, including improvements in CV and renal outcomes, and also a reduction in blood pressure and body weight as noted in large RCTs and real-world observational studies. These pleiotropic effects help prevent or reduce macro- and microvascular complications and may be particularly beneficial for patients with or at risk for diabetes-related complications, such as CVD, HF, or CKD [61]. The CV-protective effect of SGLT2i is found to be significant, regardless of the glucose-lowering effect. SGLT2i can improve the prognosis of patients after percutaneous coronary intervention (PCI) by improving glucose and lipid metabolism, inhibiting inflammatory responses and oxidative stress, and improving endothelial function and vascular remodeling. Early initiation of SGLT2i may reduce the size of MI and the occurrence of reperfusion injury during acute MI (AMI) and improve the further outcome of left ventricular dysfunction after AMI. SGLT2i are a new hope for improving the prognosis of patients with coronary artery disease, especially MI, and preventing recurrent CV events [62].

The Dapagliflozin and Prevention of Adverse-Outcomes in Heart Failure (DAPA-HF), a phase 3, placebo-controlled trial, which randomly assigned 4,744 patients with New York Heart Association class II, III, or IV HF and an ejection fraction (EF) of ≤40% to receive either dapagliflozin (at a dose of 10 mg once daily) or placebo, in addition to recommended therapy, showed that among patients with HF and a reduced EF, the risk of worsening HF or death from CV causes was lower among those who received dapagliflozin vs. those who received placebo, regardless of the presence or absence of diabetes [63]. Although one of the major concerns with SGLT2i is that it causes a pharmacologically induced glucosuria, thereby creating favorable conditions for the proliferation of pathogens that cause GTIs, these infections are generally mild to moderate in nature and respond well to conventional treatment and appropriate counseling [64].

The advantages of SGLT2i for CV health are widely recognized, while DPP4i are considered to have a neutral effect on the heart [65]. The DPP4i are not known to cause hypoglycemia or weight gain, require no dose escalation, and have favorable anti-inflammatory and safety profiles. They are relatively safe for elderly diabetic patients and patients with diabetes with kidney disease. The DPP4i have been widely used, providing substantial clinical experience. They are an excellent treatment option, and it can confidently be said that the enthusiasm surrounding DPP4i is well justified [66].

The Trial Evaluating Cardiovascular Outcomes with Sitagliptin (TECOS), a randomized, double-blind study, which included 14,671 patients with T2DM and established CVD, showed adding sitagliptin to usual care did not appear to increase the risk of MACE, hospitalization for HF, or other adverse events. During a median follow-up of 3.0 years, there was a small difference in HbA1c levels (least-squares mean difference for sitagliptin vs. placebo, −0.29 percentage points; 95% CI: −0.32 to −0.27). The CV outcome of a composite of CV death, nonfatal MI, nonfatal stroke, or hospitalization for unstable angina was 11.4%, 4.06/100 person-years with sitagliptin vs. 11.6%, 4.17/100 person-years with placebo. Sitagliptin was noninferior to placebo for the primary composite CV outcome (HR, 0.98; 95% CI: 0.88-1.09; p < 0.001) with no difference in rates of hospitalization for HF between the two groups (HR, 1.00; 95% CI: 0.83-1.20; p = 0.98) and no significant between-group differences in rates of acute pancreatitis (p = 0.07) or pancreatic cancer (p = 0.32) [67].

The FDC of dapagliflozin plus sitagliptin can be considered a suitable choice for the management of T2DM to target multiple pathophysiologies in T2DM with their complementary mechanisms of action. The combination is effective in lowering HbA1c with a low risk of hypoglycemia and weight neutrality with sitagliptin and weight loss with dapagliflozin. They help control glycemic variability throughout the day. They may be used in T2DM patients with HF with the advantage of once-daily dosing, lower risk of hypoglycemia, and CV and renal safety. The combination is useful in T2DM patients with atrial fibrillation and patients with a higher risk of hypoglycemia. The FDC of dapagliflozin and sitagliptin can be considered as an initial combination therapy in patients with T2DM with baseline HbA1c ≥ 7.5%, who are not tolerating metformin therapy, or when metformin is contraindicated. It can be used in patients with a history or risk of genitourinary-mycotic infections with patient education on genital hygiene and prompt reporting of UTI symptoms that allows for early treatment, reducing the infection burden and mortality. The incidence of genitourinary tract infections decreases with the addition of sitagliptin to dapagliflozin, as DPP4 enzymes in fungi and bacteria are inhibited by DPP4i, which is beneficial when combining DPP4i with SGLT2i. Dapagliflozin significantly reduces the risk of a decline in estimated glomerular filtration rate by at least 50%, end-stage kidney disease, or death from renal causes, and sitagliptin has a beneficial effect in preventing diabetic neuropathy, making it a valuable option for preventing diabetes-related renal complications [17].

A real-world retrospective study assessing the efficacy and safety of FDC of dapagliflozin and sitagliptin in the Indian population (DAPSI) evaluated the real-world effectiveness of the FDC in 358 patients with T2DM. The study found a significant reduction in HbA1c levels by 1.7% (reduction from 8.9 to 7.2 (p < 0.0001)), fasting blood glucose levels from 178.8 to 124.0 (p < 0.0001), and PPBG levels from 273.9 to 176.0 (p < 0.0001) after 12 weeks of treatment. The FDC of dapagliflozin and sitagliptin was found to be effective and safe in reducing blood glucose levels and BMI in the Indian population, which could help optimize treatment strategies and improve patient outcomes [68].

A real-world, retrospective, observational study evaluating the safety, efficacy, and clinical utilization of the FDC dapagliflozin plus sitagliptin in the Indian population with T2DM (SIDAXA) in 328 patients across 111 centers in India reported a significant reduction in HbA1c by 1.05% ± 0.83% (p < 0.0001) at week 12. A significant reduction was also noted in FPG by 22.98 ± 22.23 mg/dL (p < 0.0001) and PPBG by 165.50 ± 37.02 mg/dL. By week 12, significant reductions were also noted in systolic blood pressure (SBP) (14.61 ± 13.98 mmHg, p < 0.0001), diastolic blood pressure (DBP) (7.80 ± 8.45 mmHg, p < 0.0001), and low-density lipoprotein-cholesterol (LDL-C) levels (18.14 ± 23.95 mg/dL, p < 0.0001). In patients with established CVD, the reduction in HbA1c was 1.02% ± 0.63%, FPG was 54.52 ± 32.67 mg/dL, and PPBG was 88.73 ± 44.90 mg/dL after 12 weeks. The FDC of dapagliflozin plus sitagliptin was found to be a promising and well-tolerated therapeutic option for people with T2DM and established CV disease [54].

Combining dapagliflozin and sitagliptin in FDC to target multiple pathophysiological pathways for the management of T2DM appears to have several benefits, including extra-glycemic benefits due to its ability to lower blood pressure and body weight, which supports the use of this combination early in the management of T2DM [15].

Study limitations

All advisory meetings included cardiologists from Tier 1 and Tier 2 metropolitan cities and did not include specialists practicing in rural areas. This omission may have limited the perspectives on the unique challenges and realities faced in rural healthcare settings.

## Conclusions

Effective glycemic control is one of the most important strategies for the prevention of ASCVD. Earlier is better, i.e., T2DM diagnosed on time, treated on time, and treated to the goal will be more effective in delaying the onset of complications. Moving from a stepwise treat-to-failure approach to a treat-to-target approach with initial combination therapy needs to be considered in people with T2DM with HbA1c above the target and CVD risk factors. Early combination therapy can shorten the time to attain treatment goals and reduce the time to treatment failure. The combination of SGLT2i plus DPP4i has glycemic as well as nonglycemic benefits with synergistic effects on multiple pathophysiological mechanisms of T2DM. FDC of dapagliflozin and sitagliptin is a rational and synergistic combination with proven benefits in early intensive therapy in the management of people with T2DM with CVD risk.
